# Prevalence of Aflatoxin Contamination in Maize and Groundnut in Ghana: Population Structure, Distribution, and Toxigenicity of the Causal Agents

**DOI:** 10.1094/PDIS-05-17-0749-RE

**Published:** 2018-02-13

**Authors:** D. Agbetiameh, A. Ortega-Beltran, R.T. Awuah, J. Atehnkeng, P.J. Cotty, R. Bandyopadhyay

**Affiliations:** International Institute of Tropical Agriculture (IITA), PMB 5320, Ibadan, Nigeria, and Department of Crop and Soil Sciences, Kwame Nkrumah University of Science and Technology (KNUST), Kumasi, Ghana; IITA, PMB 5320, Ibadan, Nigeria; Department of Crop and Soil Sciences, KNUST, Kumasi, Ghana; Chitedze Research Station, P.O. Box 30258, Lilongwe 3, Malawi; United States Department of Agriculture, Agricultural Research Service, School of Plant Sciences, University of Arizona, Tucson, AZ 85721

## Abstract

Aflatoxin contamination in maize and groundnut is perennial in Ghana with substantial health and economic burden on the population. The present study examined for the first time the prevalence of aflatoxin contamination in maize and groundnut in major producing regions across three agroecological zones (AEZs) in Ghana. Furthermore, the distribution and aflatoxin-producing potential of Aspergillus species associated with both crops were studied. Out of 509 samples (326 of maize and 183 of groundnut), 35% had detectable levels of aflatoxins. Over 15% of maize and 11% of groundnut samples exceeded the aflatoxin threshold limits set by the Ghana Standards Authority of 15 and 20 ppb, respectively. Mycoflora analyses revealed various species and morphotypes within the Aspergillus section Flavi. A total of 5,083 isolates were recovered from both crops. The L morphotype of Aspergillus flavus dominated communities with 93.3% of the population, followed by Aspergillus spp. with S morphotype (6%), A. tamarii (0.4%), and A. parasiticus (0.3%). Within the L morphotype, the proportion of toxigenic members was significantly (P < 0.05) higher than that of atoxigenic members across AEZs. Observed and potential aflatoxin concentrations indicate that on-field aflatoxin management strategies need to be implemented throughout Ghana. The recovered atoxigenic L morphotype fungi are genetic resources that can be employed as biocontrol agents to limit aflatoxin contamination of maize and groundnut in Ghana.

This study was funded by the Meridian Institute (9678.3 IITA (BC) 2012) on behalf of Partnership for Aflatoxin Control in Africa (PACA), the Bill & Melinda Gates Foundation (OPP1133356), and the USDA-Foreign Agricultural Service (58-3148-2-246). This work was undertaken as part of the CGIAR Research Program on Agriculture for Nutrition and Health (A4NH).

Maize (Zea mays L.) and groundnut (Arachis hypogaea L.) are staple crops for billions across the globe (FAOSTAT 2016). However, in warm agricultural areas, both crops are frequently infected by Aspergillus section Flavi fungi (Samson et al. 1981) and contaminated with aflatoxins before, during, and after harvest (Bandyopadhyay and Cotty 2013; Cotty et al. 1994). Aflatoxins are highly toxic and carcinogenic compounds that negatively impact the health of both humans and livestock (Bryden 2012; Wild and Gong 2010). Chronic and/or acute effects, including death, may result after consumption of afatoxin-contaminated commodities (Gong et al. 2008; Probst et al. 2007). Aflatoxin contamination of various crops also impacts trade and economic growth. Commodities exceeding aflatoxin thresholds cannot enter premium markets, particularly in developed countries where aflatoxins are strictly monitored and regulated (van Egmond et al. 2007; Wu 2004, 2015).

The most common aflatoxin-producing species worldwide is Aspergillus flavus Link (Klich 2007). This species is subdivided into two distinct morphotypes, L and S, which vary widely in morphology, epidemiology, and physiology, including their potential to produce aflatoxins (Cotty 1989; Mehl et al. 2012). The L morphotype produces fewer, larger sclerotia (avg. diameter > 400 µm), numerous conidia, and variable levels of aflatoxins, while the S morphotype produces abundant small sclerotia (avg. diameter < 400 µm), few conidia, and consistently high levels of aflatoxin (Cotty 1989). The S morphotype often constitutes a minor proportion of the aflatoxigenic communities associated with a crop but is considered an important causal agent of contamination because of its capability to produce high aflatoxin levels (Cardwell and Cotty 2002; Jaime-Garcia and Cotty 2010; Probst et al. 2010). Fungal examination of toxigenic communities across the globe has revealed that there are several lineages of fungi with S morphotype with some of them producing large concentrations of both B and G aflatoxins. For instance, in West Africa, there is a group of fungi with S morphotype that uniformly produces both B and G aflatoxins and known as unnamed taxon SBG (Atehnkeng et al. 2008; Cotty and Cardwell 1999; Diedhiou et al. 2011; Perrone et al. 2014; Pildain et al. 2008; Probst et al. 2014).

Certain genotypes of the A. flavus L morphotype are atoxigenic (i.e., do not produce aflatoxins) as a result of genetic defects in one or several genes necessary for aflatoxin production (Chang et al. 2005; Donner et al. 2010). Atoxigenic L morphotype genotypes are relatively common in any given area (Atehnkeng et al. 2008; Cotty 1997; Jiang et al. 2009; Mauro et al. 2015; Ortega-Beltran et al. 2016; Probst et al. 2011; Zanon et al. 2013), while atoxigenicity in S morphotype genotypes has been reported only once in an isolate collected in the U.S. (Horn and Dorner 1999).

In Ghana, maize and groundnut are major staple crops for the majority of the population (MoFA 2011). Maize is produced in all agroecological zones (AEZs) within Ghana, mostly under rainfed conditions, and primarily by smallholder farmers (Agyare et al. 2014). More than 90% of groundnut production in Ghana is concentrated in the Northern, Upper East, and Upper West regions, which lie within the Southern Guinea Savanna (SGS) and Derived Savanna (DS) AEZs (MoFA 2011), where the crop is an important source of revenue for many farmers.

Consumption of both maize and groundnut results in high human aflatoxin exposure in Ghana. However, most Ghanaians have little to no knowledge of either what aflatoxins are or the health risks posed by these toxins (Awuah et al. 2008). Aflatoxin awareness is low in spite of frequent reports of aflatoxin contamination of foods for over five decades in Ghana (Beardwood 1964; Kpodo et al. 1996; Kumi et al. 2014; Mintah and Hunter 1978) and surveys describing aflatoxin toxicological effects in Ghanaians, particularly among women and children (Afum et al. 2016; Apeagyei et al. 1986; Jiang et al. 2005; Jolly et al. 2006; Lamplugh et al. 1988; Shuaib et al. 2012). The trade sector has been affected as well. Commodities from Ghana (peanut butter, spices, and edible seeds) exceeding tolerance thresholds have been rejected in European borders (RASFF 2017). As a consequence, Ghana faces a threat of an export ban of aflatoxin-susceptible commodities if necessary actions to reduce aflatoxin levels in trade commodities are not taken (Dzirasah 2015). Trade restrictions have affected several other African nations due to high aflatoxin crop content, resulting in severe economic losses (Bandyopadhyay et al. 2016; RASFF 2017).

Many technologies limit crop aflatoxin contamination. These include good agricultural practices, monitoring and crop destruction, and postharvest interventions (Florkowski and Kolavalli 2013; Hell et al. 2008; Jaime-Garcia and Cotty 2004; Jones 1987). Because the aflatoxin contamination process often starts in the field, interventions are needed before crop maturity (Cotty 2006; Dorner 2004). In Ghana, efforts to reduce aflatoxin contamination of maize and groundnut have been directed to postharvest strategies and improvement of storage conditions (Florkowski and Kolavalli 2013). Such interventions may significantly decrease further aflatoxin accumulation after harvest but will not impact preharvest aflatoxin contamination in the field.

The most effective and proven method for prevention of aflatoxin contamination is to use atoxigenic strains of A. flavus as biocontrol agents to decrease the proportion of toxigenic fungi in treated fields (Bandyopadhyay et al. 2016; Wu et al. 2008). This results in significantly less aflatoxin concentrations in crops from treated fields in comparison with crops from untreated fields grown in the same area, in the same cropping season. In the U.S., hundreds of thousands of hectares are treated annually with biocontrol agents and this allows production of crops meeting aflatoxin standards (Cotty 2006; Dorner 2004, 2009; Doster et al. 2014). Nigeria, Kenya, and Senegal are African nations that have successfully adopted aflatoxin management strategies by employing native atoxigenic strains as biocontrol agents; other African nations are in the process of developing this strategy (Bandyopadhyay et al. 2016). Adoption of aflatoxin biocontrol technologies in Ghana for use in maize and groundnut would potentially reduce both aflatoxin concentrations in these crops and consequently human exposure to the toxins. However, implementation of aflatoxin biocontrol technologies within Ghana is dependent on identifying atoxigenic isolates among communities of aflatoxin-producing fungi associated with both maize and groundnut produced in Ghana. Use of native atoxigenic strains adapted to local agroecosystems ensures a greater displacement of aflatoxin-producing strains in comparison with exotic atoxigenic strains that may lack adaptation to compete for local resources (Mehl et al. 2012; Probst et al. 2011). In addition, use of native atoxigenic strains have greater acceptance by regulatory agencies (Bandyopadhyay et al. 2016).

The population structure, distribution, and toxigenicity of aflatoxin-producing fungi associated with maize and groundnut in Ghana remains to be investigated. In addition, little is known about which areas within Ghana are hotspots for aflatoxin contamination of maize and groundnut. Generating knowledge of the interaction of aflatoxin-producing fungi with both maize and groundnut in major producing areas of Ghana is critical to designing aflatoxin management strategies. Therefore, the objectives of this study were to i) determine the prevalence of aflatoxin contamination in maize and groundnut from major producing AEZs in Ghana, ii) assess the population structure and distribution of the etiologic agents of aflatoxin contamination across three AEZs, and iii) determine aflatoxin-producing potentials of communities residing within three AEZs to identify atoxigenic strains of A. flavus that can be used in aflatoxin biocontrol programs. Our results highlighted the extent of aflatoxin contamination in maize and groundnut in Ghana and identified areas prone to contamination. In addition, a large germplasm collection of atoxigenic A. flavus L morphotype isolates was identified. These native atoxigenic strains of A. flavus constitute genetic resources with the potential for use as biocontrol agents to reduce aflatoxin contamination in maize and groundnut throughout Ghana.

## Materials and Methods

**Fig. 1:**
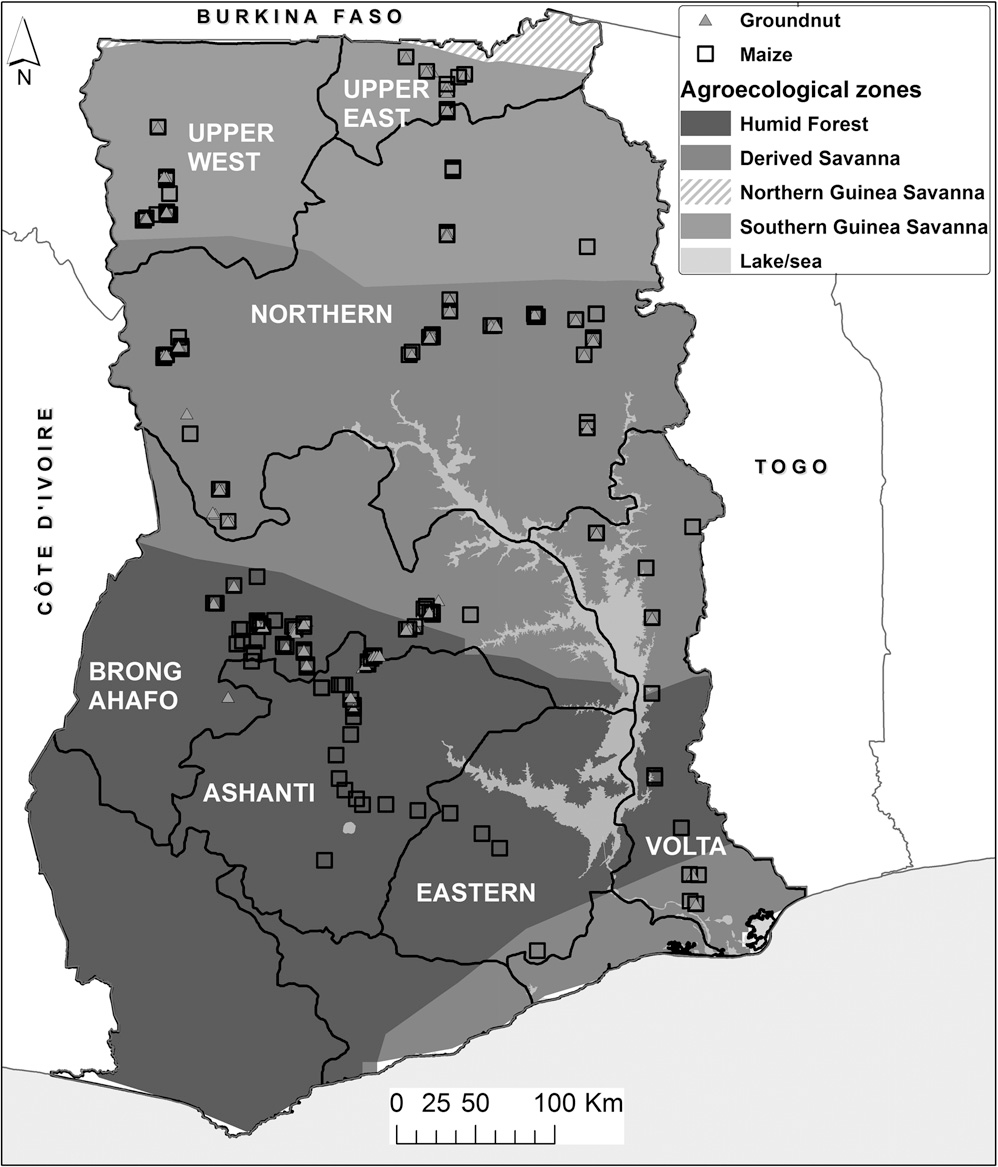
Map of Ghana depicting agroecological zones and the seven regions where samples were collected. The Southern Guinea Savanna (SGS) zone has a semiarid climate, a unimodal rainfall pattern of up to 1,100 mm, and a growing period of 180 to 200 days. Poor soils low in organic matter with high levels of iron and susceptible to severe erosion are common in SGS. The Derived Savanna (DS) zone has a subhumid climate, an annual precipitation range of 1,000 to 1,500 mm, and a growing period of 180 to 270 days. Soils in DS are principally sandy loams to clay loams, and are mostly poorly drained. The Humid Forest (HF) zone is characterized by a bimodal rainfall pattern ranging from 1,500 to 2,200 mm and a growing period of 270 to 365 days. Soils in HF are generally loamy, porous, well drained, and have greater accumulation of organic matter in the topsoil in comparison with SGS and DS (MoFA 2011; Oppong-Anane 2006)

Area of study. Maize and groundnut are produced primarily within seven regions of Ghana spanning three AEZs: the SGS, the DS, and the Humid Forest (HF). Figure 1 provides information on the location of sampling sites.

Minor and major cropping seasons. The bimodal rainfall pattern in the HF and the southern parts of the DS enables two cropping seasons: the major and minor seasons. The major season is characterized by heavy precipitation from April to July, followed by a humid period in August (Nkrumah et al. 2014). The minor cropping season begins from September to November and is followed by the Harmattan season from December to March. The Harmattan season is a dry period in which dust blown from the Sahara desert reaches large portions of West Africa, including Ghana (Oppong-Anane 2006). On the other hand, the northern part of the DS encompassing the Northern region and the entire SGS (Upper East and Upper West regions) is characterized by a unimodal rainfall pattern, allowing for only one growing season (major season) occurring between May and November (Nkrumah et al. 2014).

Sample collection and preparation. Maize and groundnut samples were collected from farmers’ storage structures during March of 2013 in seven major producing regions of Ghana (Fig. 1). Samples belong to crops planted during either the major or minor cropping season of 2012, depending on the region and AEZ sampled. Shelled or in-shell groundnuts stored for 2 to 5 months were collected from jute or interlaced polypropylene bags, and in-earth cribs. Maize samples consisted of shelled, husked, or de-husked maize. While shelled maize was stored in interlaced polypropylene bags, husked or dehusked maize ears were stored on raised platforms, wooden cribs, as heaps on bare or cemented floor, or spread on tarpaulin. Representative samples (0.5 to 1 kg) were collected from each storage type. A total of 509 samples, comprising 326 maize and 183 groundnut samples, were collected. Following phytosanitary certification of the samples by the Plant Protection and Regulatory Services Directorate of Ghana’s Ministry of Food and Agriculture, all samples were transported to the Pathology and Mycotoxin laboratory of the International Institute of Tropical Agriculture (IITA) for laboratory analyses under import permits issued by Nigeria Agricultural Quarantine Service. Immediately after arrival at the laboratory, ears of maize and podded groundnut samples were shelled manually. Samples were thoroughly mixed and a half portion was blended using a laboratory blender (Waring Commercial, Springfield, MO) for 30 s in a 110-ml stainless steel blending jar (MC-2) and stored at 4°C before aflatoxin and microbial analyses. The blending jar was washed between samples with 80% ethanol to prevent cross contamination by both microorganisms and aflatoxins.

Aflatoxin analysis. Aflatoxins were extracted from maize by combining 20 g ground samples with 100 ml 70% methanol (Atehnkeng et al. 2008). For groundnut, 20 g ground samples were combined with 100 ml 80% methanol (Cole and Dorner 1993). Suspensions were shaken on a Roto-Shake Genie (Scientific Industries, Bohemia, NY) for 30 min at 400 rpm and filtered through Whatman No. 1 filter paper (Whatman International Ltd., Maidstone, England). Filtrates were collected in 250 ml separatory funnels, combined with 100 ml distilled water, and extracted twice with 25 ml methylene chloride. The methylene chloride phase was filtered through a bed of 25 g anhydrous sodium sulfate contained in fluted Whatman No. 4 filter paper, combined, and evaporated to dryness in a fume hood (Cotty and Cardwell 1999). Residues were dissolved in 1 ml methylene chloride and subjected to scanning densitometry. Homogenates were directly spotted (4 µl) alongside aflatoxin standards (Supelco, Bellefonte, PA) on thin layer chromatography (TLC) aluminum (20 × 10 cm) silica gel 60 F254 plates (Merck, Darmstadt, Germany) and developed with diethyl ether-methanol-water (96:3:1) (Cotty 1997; Probst et al. 2011). Plates were visualized under ultraviolet light (365 nm) for presence or absence of aflatoxins. Aflatoxins were quantified directly on TLC plates with a scanning densitometer (CAMAG TLC Scanner 3) and quantification software (winCATS 1.4.2, Camag, AG, Muttenz, Switzerland).

Fungal isolation and characterization. Structures of fungal communities associated with maize and groundnut were determined by assigning isolates within Aspergillus section Flavi to their corresponding species and/or morphotypes. Isolates were obtained from each sample by dilution plate technique on modified Rose Bengal agar (MRBA) (Cotty 1994). Briefly, 1 g of each sample was suspended in 10 ml sterile distilled water contained in a 40 ml sterile polystyrene tube, vortexed for 1 min, and plated on MRBA at appropriate dilutions to ensure the recovery of no more than 10 Aspergillus section Flavi colonies per plate. Plates were incubated for 3 days (31 °C, dark). Colonies of Aspergillus section Flavi were identified by macroscopical morphology (Cotty 1989; Klich and Pitt 1988). For each sample, 10 isolates were transferred to 5-2 agar (5% V-8 juice [Campbell Soup Company, Camden, NJ], 2% bacto agar [Difco Laboratories Inc., Detroit, MI], pH 6.0) and incubated in the dark for 5 days at 31 °C for further characterization. Members of Aspergillus section Flavi were identified based on their colony characteristics and spore ornamentation and classified into species and morphotypes. Within A.flavus, isolates with smooth conidia and large sclerotia (avg. diameter > 400 µm) were classified as L morphotype (Cotty 1989); those with numerous small sclerotia (avg. diameter < 400 µm) were classified as having the S morphotype. A. parasiticus and A. tamarii were identified by colony color and spore ornamentation (Klich and Pitt 1988). Incidences of Aspergillus section Flavi species in maize and groundnut samples were calculated as colony-forming units (CFU) per g of sample. A total of 5,083 isolates were recovered and maintained as agar plugs (3-mm diameter) of sporulating cultures in 2 ml sterile distilled water as working cultures, and on silica gel at 4°C for long term storage as described by Probst et al. (2011).

In vitro aflatoxin production and analytical determination. The aflatoxin-producing ability of each of the 5,083 isolates of Aspergillus section Flavi was evaluated in sterile, dead-autoclaved maize kernels. Maize was selected as a substrate to examine aflatoxin-producing potentials because true potentials are revealed using a living substrate in comparison with chemical defined media (Probst and Cotty 2012). Five grams of healthy maize grains from a maize batch previously known to be free of aflatoxins were weighed into 40 ml polystyrene glass vials and washed with two exchanges of tap water to remove surface contaminants. This was followed by soaking with 25 ml tap water overnight to allow imbibing adequate moisture for fungal growth. The next day, water was decanted, kernels rinsed with two exchanges of tap water, and water was decanted again. Kernels were sterilized by autoclaving at 121 °C for 20 min. After autoclaving, kernels were allowed to reach room temperature under aseptic conditions. The sterilized, moistened kernels were independently inoculated with 500 ml of a suspension containing approximately 106 spores of each isolate and incubated at 31 °C for 7 days in the dark. Kernels inoculated with 500 µl of sterile distilled water served as negative control. Aflatoxins were extracted from maize fermentations and quantified as described above. Those maize fermentations from which aflatoxins were not initially detected were extracted, concentrated, and quantified as above. Isolates that did not produce aflatoxins on maize fermentations were classified as atoxigenic. The limit of detection of aflatoxin B1 was 0.1 parts per billion (ppb).

Data analysis. Maize and groundnut samples were grouped into four categories based on their aflatoxin content. The categories were assigned based on aflatoxin limits imposed by the European Union (4 ppb), the Ghana Standards Authority (GSA; 15 ppb for maize and 20 ppb for groundnut), and the United States Food and Drugs Administration (FDA; 20 ppb) as follows: i) samples with no detectable aflatoxins, ii) <4 ppb, iii) <15 (for maize) or <20 ppb (for groundnut), and iv) ≥15 (for maize) or ≥20 ppb (for groundnut).

Values for fungal type incidence, densities, aflatoxin-producing potentials of the recovered fungi, and atoxigenic fungi frequencies in all AEZs, were subjected to analysis of variance (ANOVA) with the general linear model (GLM) suitable for unbalanced data. All statistical tests were performed with SAS (version 9.4, SAS Institute Inc., Cary, NC). The GLM of SAS uses the least-squares method to fit data to a general linear model. Means were separated with the Fisher’s protected least significant difference (LSD) test at 5% significance level. Values for fungal incidences were arcsine square root transformed while values for both fungal densities and aflatoxin-producing potentials were log transformed prior to analysis to normalize the variance. In the tables, nontransformed data are presented.

## Results

Prevalence of aflatoxin contamination in maize and groundnut in Ghana. A majority of the maize and groundnut samples in this study were not contaminated with aflatoxins beyond the threshold limit set by GSA (Table 1). Nevertheless, 15.3% of maize samples and 11.5% of groundnut samples exceeded the regulatory limit set by GSA with some maize samples exceeding 22 times and groundnut samples 190 times the GSA limits for human consumption. In general, the aflatoxin contamination level was lower in maize (mean: 11 ppb; range: not detectable to 341 ppb) than in groundnut (mean: 58 ppb; range: not detectable to 3,868 ppb) (Table 1).

**Table 1:** Percent and numbers of maize and groundnut grain samples from Ghana with different levels of aflatoxin concentration

Category of aflatoxin concentration (ppb)^x^	Percent (number) of crop samples^y^	
	Groundnut	Maize
ND	63.4 (116)	62.3 (203)
<4	80.9 (148)	70.0 (228)
<15	N/A^z^	84.7 (276)
≥15	N/A	15.3 (50)
<20	88.5 (162)	N/A
≥20	11.5 (21)	N/A

x ND = Not detected; limit of detection = 0.1 ppb. <4 = European Union standard; <20 (groundnut) and <15 (maize) = Ghana Standard Authority standard; ≥20 (groundnut) or ≥15 (maize) = Not fit for human consumption in Ghana.

y Percent of crop samples belonging to the indicated category is listed first. Numbers in parenthesis represent number of samples in the indicated category.

z N/A = not applicable; aflatoxin tolerance threshold for groundnut in Ghana is 20 ppb. Therefore, the categories of <15 and ≥15 were not considered for this crop. Similarly, for maize, the tolerance threshold is 15 ppb, and hence the categories of <20 and $20 were not considered for this crop.

Aflatoxin concentrations in some AEZs were above tolerance thresholds (Table 2). In maize, mean aflatoxin concentration in DS and SGS was >15 ppb, except in the Eastern region (DS) and Northern region (SGS). Aflatoxin concentration in groundnut from the Volta region (HF), Brong Ahafo and Northern regions (DS), and Northern (SGS), on average, exceeded 20 ppb. Some groundnut samples from the Northern region (DS) contained nearly 4,000 ppb total aflatoxins (Table 2). There was no correlation between the types of storage and aflatoxin accumulation (data not shown).

**Table 2:** Mean and range of aflatoxin concentration (ppb) in stored maize and groundnut samples collected from seven major producing regions in three agroecological zones (AEZs) in Ghana in March 2013

		Maize (aflatoxin, ppb)			Groundnut (aflatoxin, ppb)		
AEZ^w^	Region^x^	*n*^y^	Mean	Range	*n*^y^	Mean	Range
HF	Ashanti	52	6.1	0–135	12	2.2	0–17
	Brong Ahafo	50	0.6	0–9	16	5.5	0–54
	Eastern^z^	4	1	0–3	-	-	-
	Volta	29	9	0–83	11	42.4	0–387
DS	Brong Ahafo	20	16.8	0–226	27	145.6	0–1,999
	Eastern^z^	1	0	0	-	-	-
	Northern	90	15.9	0–341	72	77.7	0–3,868
	Volta	8	24.2	0–157	2	0.3	0–1
SGS	Northern	14	6.8	0–59	5	34.9	0–168
	Upper East	15	15.4	0–82	14	0.3	0–1
	Upper West	43	16.4	0–190	24	15.9	0–181

w HF = Humid Forest, DS = Derived Savanna, and SGS = Southern Guinea Savanna.

x Samples were collected in regions within different AEZs. For example, in Brong Ahafo, samples from both the HF and DS were collected.

y **n** = Number of maize/groundnut samples collected and tested for aflatoxin content.

z Groundnut was not cropped/stored in the sampled areas of the Eastern region at the time of the study.

Population structure and distribution of Aspergillus section Flavi. Aflatoxin-producing fungi were detected in all examined samples. A total of 5,083 isolates (3,260 from maize and 1,823 from groundnut) belonging to various species and morphotypes within Aspergillus section Flavi were recovered. The recovered fungi included the A. flavus L morphotype, fungi with S morphotype, A. parasiticus, and A. tamarii. Across the three AEZs, the L morphotype largely dominated the examined communities followed by the S morphotype. Incidences of both A. parasiticus and A. tamarii were rare and inconsistent. A. parasiticus was not detected in SGS. No significant differences (P = 0.05) were detected in species or morphotype incidences across AEZs. Significantly (P < 0.05) lower fungal densities were detected in HF than in both DS and SGS (Table 3). Further, higher fungal densities were detected in maize than in groundnut and this was significantly (P < 0.05) higher in DS and SGS (Table 3).

**Table 3:** Incidence and densities of Aspergillus section Flavi in maize and groundnut samples across three agroecological zones (AEZs) in Ghana

			Incidence (%)^z^				CFU/g	
AEZ^x^	Crop	n^y^	L	S	Ap	At	Mean	Range
HF	Maize	1,350	95.3 a	4.4 a	0.0 b	0.3 a	11,819 a	4–500,000
	Groundnut	383	92.1 a	6.9 a	0.5 a	0.5 a	2,238 a	2–40,000
DS	Maize	1,190	95.0 a	4.7 b	0.0 b	0.3 a	51,940 a	5–105,000
	Groundnut	1,010	88.1 b	10.6 a	1.0 a	0.3 a	15,122 b	2–666,667
SGS	Maize	720	93.1 a	6.5 a	0.0 a	0.4 a	36,428 a	5–540,000
	Groundnut	430	94.6 a	4.2 a	0.0 a	1.2 a	2,206 b	2–36,000

x HF = Humid Forest, DS = Derived Savanna, and SGS = Southern Guinea Savanna.

y *n* = Number of isolates examined.

z Within an AEZ, incidence of each fungus type (L = A. *flavus* L morphotype, S = isolate with S morphotype, Ap = A. *parasiticus*, At = A. *tamarii*) is compared between crops. For example, in HF, frequencies of the L morphotype were not significantly different in maize and groundnut. Means with the same letter are not significantly different according to Fisher’s least significant difference test (α = 0.05). Values for fungal incidences were arcsine square root transformed prior to analyses. Values for fungal densities were log transformed prior to analysis.

The L morphotype was consistently the most common Aspergillus group associated with both maize and groundnut. DS was the sole AEZ in which incidences of L and S morphotypes differed (P < 0.05) between maize and groundnut (Table 3). Similar incidences of isolates with S morphotype were detected in maize and groundnut in both HF and SGS. A. parasiticus was not detected on maize in any AEZ nor in groundnut from SGS.

**Fig. 2:**
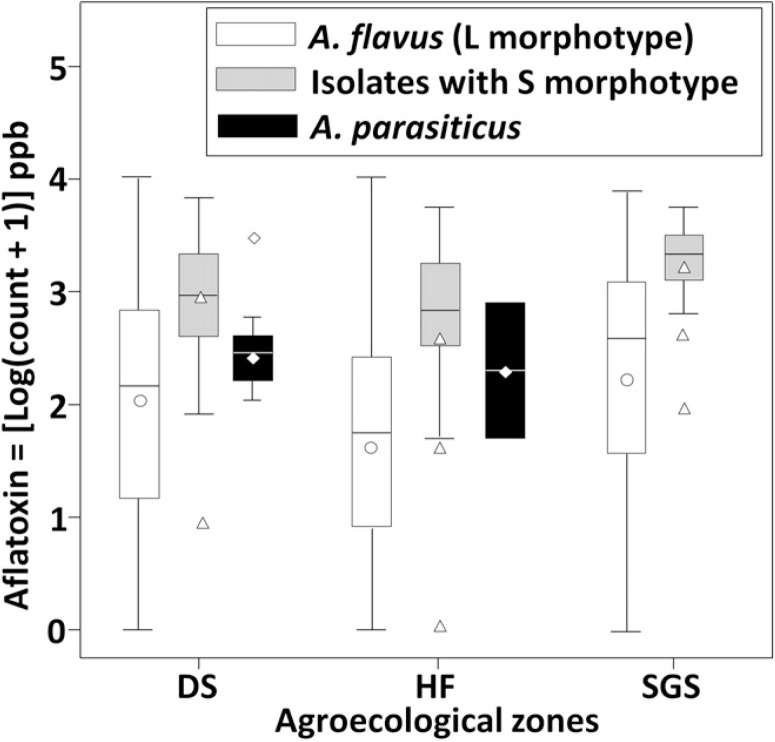
Box plot showing the variation in quantities of aflatoxin B1 produced by isolates of *Aspergillus flavus* L morphotype, isolates with S morphotype, and *A. parasiticus* across three agroecological zones (AEZs) in Ghana. Boxes indicate the interquartile range of aflatoxin B_1_ produced by each type of fungi within an AEZ. Solid lines and symbols inside boxes represent the median and means, respectively. Whisker bars indicate the 10th and 90th percentiles while points below or above whisker bars are outliers. DS = Derived Savanna; L morphotype = 2,020 isolates, S morphotype = 160 isolates, *A. parasiticus* = 13 isolates. HF = Humid Forest; L morphotype = 1,639 isolates, S morphotype = 86 isolates, *A. parasiticus* = 2 isolates. SGS = Southern Guinea Savanna; L morphotype = 1,077 isolates, S morphotype = 65 isolates.

Aflatoxin production in maize kernels. The causal agents of aflatoxin contamination in Ghana varied in their potential to produce B and G aflatoxins when inoculated in dead autoclaved maize kernels (Table 4). From the toxigenic fungi, all A. flavus L morphotype isolates produced only B aflatoxins. Mean aflatoxin-producing abilities of this morphotype varied significantly (P < 0.05) across the three AEZs (Table 4), with isolates from SGS producing the highest concentrations. When comparing aflatoxin-producing abilities among types of fungi, significant (P < 0.05) differences were detected in each AEZ (Fig. 2, Table 4).

**Table 4:** Aflatoxin-producing potential of Aspergillus section Flavi isolates in three agroecological zones (AEZs) of Ghana

Aflatoxin concentration (ppb)^z^									
	A. flavus L morphotype			S morphotype			A. parasiticus		
AEZ^^x^^	n^^y^^		B_1_	n^y^	B_1_	G_1_	n^y^	B_1_	G_1_
HF	1,648	Mean^z^	299 bC	86	1,193 aB	2,706 aA	2	492 bA	159 bA
		Range	0–16,673		0-7,689	0–17,772		36–948	19–298
DS	2,010	Mean	518 bB	160	1,417 aA	1,852 aC	13	473 bA	542 bA
		Range	0–11,429		11–7,935	19–10,692		111–2,843	133–2,360
SGS	1,078	Mean	727 bA	65	1,836 aA	2,545 B	-	-	-
		Range	0–8,548		71–6,885	125–9,282		-	-

x HF = Humid Forest, DS = Derived Savanna, SGS = Southern Guinea Savanna.

y n = Number of isolates.

z Values with the same letter are not significantly different according to Fisher’s least significant difference (LSD) test (a = 0.05). Lower case letters compare either aflatoxin B_1_ or G_1_ (rows) production within AEZs. Upper case letters compare toxin-production ability of each type of fungi across AEZs (columns). B_1_ and G_1_ toxins are compared independently. Values for aflatoxin-producing potentials were log transformed prior to analysis.

Over 97% of the isolates with S morphotype produced large amounts of both B and G aflatoxins. The remaining isolates (nine total and obtained from a single maize sample) produced no aflatoxins (atoxigenic). The mean aflatoxin B1 production by isolates with S morphotype was significantly (P < 0.05) higher in SGS and DS than in HF (Table 4). In addition, the majority (76%) of isolates with S morphotype, uncharacteristically, produced larger concentrations of aflatoxin G1 than of aflatoxin B1 in all three AEZs. All A. parasiticus isolates produced both B and G aflatoxins and their aflatoxin-producing potentials did not differ between the two AEZs (HF and DS) only where this species occurred. None of the A. tamarii isolates produced detectable amounts of aflatoxins (Table 4). Within AEZs, all aflatoxin-producing types of fungi (i.e., A. flavus L morphotype, A. parasiticus, and isolates with S morphotype) varied largely in their abilities to produce aflatoxin B1. Isolates with S morphotype produced significantly (P < 0.05) higher amounts of aflatoxin B1 than A. parasiticus and L morphotype in DS and SGS but no significant differences were observed among the three taxa in HF. Across AEZs, the average aflatoxin B1 producing potential of each type of fungi was higher in SGS, followed by DS, and the least in HF (Fig. 2).

**Fig. 3:**
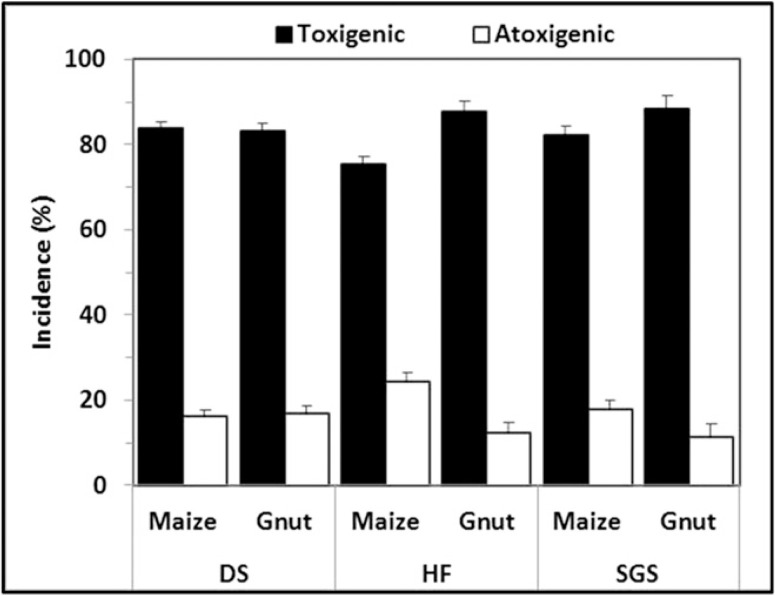
Distribution of toxigenic and atoxigenic *A. flavus* L morphotype fungi in maize and groundnut (Gnut) across three AEZs of Ghana. For each bar, vertical lines represent the standard error of the means.

Distribution of toxigenic and atoxigenic isolates of A. flavus L morphotype. Spatial distribution of toxigenic and atoxigenic L morphotype isolates varied across all three AEZs. Toxigenic isolates were significantly (P < 0.01) more prevalent than atoxigenic isolates (Fig. 3). In all, 17.8% of the tested isolates produced undetectable amounts of aflatoxins and were classified as atoxigenic. The proportion of toxigenic L morphotype members across all three AEZs was >70%. However, significantly higher (P < 0.05) proportions of toxigenic members were observed in SGS and DS in comparison with HF (Fig. 3). Frequencies of toxigenic L morphotype isolates were significantly (P < 0.01) higher in groundnut than in maize (Fig. 3).

## Discussion

We document the first extensive study on the occurrence and toxigenicity of aflatoxin-producing species associated with maize and groundnut in major producing areas of Ghana. The causal agents of aflatoxin contamination and the extent of contamination in maize and groundnut across Ghana were determined. Over 35% of maize and groundnut samples were contaminated with aflatoxins, although concentrations varied among and within AEZs. Our results indicate that both crops are at high risk for aflatoxin contamination with 15.3% of maize and 11.5% of groundnut samples exceeding GSA tolerance thresholds (GSA 2001, 2013). Indeed, the mean aflatoxin level in groundnut was nearly three times the GSA threshold. Given the dietary importance of these crops coupled with the fact that the sampled regions are major grain market outlets, it is very likely that most Ghanaians are frequently exposed to aflatoxins (Afum et al. 2016; Jolly et al. 2007; Kumi et al. 2014; Shuaib et al. 2012).

Pre- and postharvest practices largely dictate the extent to which Aspergillus fungi invade seeds and exacerbate aflatoxin production (Cotty 2006; Hell et al. 2008). Insect damage and drought stress prior to harvest favors aflatoxin formation in maize (Hell et al. 2008). Upon maturity, groundnut is dug from the soil and turned upside down haphazardly in a windrow (random windrowing) for curing/ drying in the field (Marfo et al. 2000; Waliyar et al. 2015a). This practice often exposes the crop to warm and moist conditions, which predisposes it to Aspergillus infection and subsequent aflatoxin production (Cotty and Jaime-Garcia 2007). Further, high aflatoxin levels can occur if rodents and other pests attack and damage pods, and if storage occurs under unfavorable conditions over long periods (Waliyar et al. 2015a, b). On-field drying processes following harvesting along with suboptimal storage conditions possibly influenced the high levels of aflatoxins in groundnut (Table 2). Crop drying under appropriate conditions (e.g., using a mechanical drier to rapidly achieve safe storage moisture content) would have prevented exposure of pods/kernels to moist and/or humid conditions resulting in both reduced pod invasion of aflatoxin-producing fungi and aflatoxin accumulation (Chiewchan et al. 2015; Waliyar et al. 2015b). In areas where the two seasons exist, maize grown during the minor season was collected from farmers’ stores. That maize was harvested in December 2012 and stored for around 2 months prior to sampling. The hot Harmattan conditions allow fast and proper drying of the crop in the field prior to harvest and storage. This, together with the short storage period, likely limited aflatoxin concentrations in sampled maize from the minor season (Table 2).

In SGS and a large part of DS (encompassing large areas of the three northern regions; Fig. 1), there is only one cropping season that fits into the category of major cropping season and is characterized by erratic rainfall from May to October. A severe dry period follows until the next season. This dry period allows for fast and proper drying of crops. However, farmers providing samples expressed that prolonged crop storage periods (up to 5 months) are common practice. In Mali, groundnut stored for periods of up to 3 months, in sub-optimal conditions, contained high aflatoxin concentrations (Waliyar et al. 2015b). This is expected to occur in susceptible crops stored for similar periods under related conditions in countries within the subregion (Kpodo and Bankole 2008). Indeed, in the current study, we detected high aflatoxin levels in those crops stored for up to 5 months (Table 2) and those crops were associated with higher frequencies of toxigenic fungal types (data not shown). Crop storage under sub-optimal conditions results in high levels of aflatoxins if the crop is associated with highly toxigenic fungi (Atehnkeng et al. 2008; Kachapulula et al. 2017).

Nearly four decades ago, Mintah and Hunter (1978) reported high aflatoxin levels in groundnut produced in the Northern and Volta regions that were sold in various markets in Ghana’s capital, Accra. It is not clear whether those samples became contaminated with aflatoxins before, during, or after harvest in the producing areas, or during transportation and storage prior to commercialization. In the current study, high aflatoxin levels were detected in both maize and groundnut from the Northern and Volta regions; therefore, it can be hypothesized that samples from the Mintah and Hunter study were indeed contaminated with aflatoxins in the areas in which groundnut were produced. However, the contamination process could have continued during transport and/or storage prior to commercialization. In addition, maize from the Upper East and Upper West (SGS) and groundnut from the Upper West (SGS) and Brong Ahafo (DS) regions had similarly high aflatoxin levels. Our results suggest that the indicated five regions within these AEZs, which constitute major crop-producing areas and market outlets of Ghana, are prone to aflatoxin contamination. Highly contaminated maize and/or groundnut from these regions could reach major markets and consequently homes in Ghana. Efforts targeted at mitigating aflatoxin accumulation of both crops should be primarily designed to be implemented in these regions.

Fungal densities in crops were quantified on a selective medium (MRBA), which allows for maximum detection of Aspergillus section Flavi colonies (Cotty 1994). Higher (P < 0.05) fungal quantities (CFU/g) were obtained in DS (mean = 35,037) and SGS (mean = 23,632) than HF (mean = 9,672) (Table 3). From November to March, mean monthly temperatures in DS and SGS usually exceed 25°C, which is an optimum condition for infection and growth of A. flavus (Diener et al. 1987). Thus, inoculum density was expected to be higher in crop samples collected from DS and SGS, which possess a relatively hotter and drier climate than HF. Airborne propagules of Aspergillus section Flavi are the predominant source of primary inoculum for infection of maize while in groundnut the primary source originates from the soil (Wilson and Payne 1994).

In groundnut, the pod walls serve as a physical barrier to infection when kernels are stored in-shell (Nigam et al. 2009). In maize, however, removal of the husk breaks the physical barrier and possibly further exposes the grains to the vagaries of the atmosphere, allowing direct contact and subsequent infection by airborne Aspergillus propagules (Wilson and Payne 1994). Most of the groundnut samples collected for our studies were stored in-shell while most of the collected maize was stored in de-husked form. Those practices are thought to have had a large influence on the variation in propagule densities observed between crop types across AEZs (Table 3). Rapid and adequate drying of groundnut in-shell and maize ears coupled with appropriate storage conditions are crucial for reducing fungal infection and postharvest aflatoxin accumulation.

Communities of Aspergillus section Flavi consist of a complex assemblage of individuals that vary widely in their phenotypic and genotypic characteristics (Cotty et al. 1994; Mehl and Cotty 2010) and represent an important factor in incidences and severities of contamination (Probst et al. 2010). In this study, four types of fungi within Aspergillus section Flavi were identified across three AEZs in Ghana using morphological (colony characteristics and spore ornamentation) and physiological (aflatoxin-producing profile) criteria (Klich and Pitt 1988). Isolates of A. flavus L morphotype dominated all three AEZs (Table 3). However, structures of aflatoxin-producing fungal communities vary among and within AEZs across years (Ortega-Beltran et al. 2015). Fungal community compositions reported in the current study are expected to fluctuate among AEZs in different years.

A community structure similar to the one described in the current study was reported by Perrone et al. (2014) on maize grains from markets and farms at harvest in Ghana and Nigeria. In that study, the L morphotype and a high (>70%) proportion of atoxigenic isolates dominated in samples from Ghana. However, we detected relatively low (17.8%) frequencies of atoxigenic L morphotype isolates across AEZs (Fig. 3). The use of a living substrate (Probst and Cotty 2012), as used in our study, provides true estimates of aflatoxin-production potential of isolates than chemically defined media as used by Perrone et al. (2014). Occurrence of atoxigenic isolates was expected because atoxigenic isolates have been identified in all regions where aflatoxin-producing communities have been investigated (Abbas et al. 2011; Atehnkeng et al. 2008; Cotty 1989; Mauro et al. 2015; Ortega-Beltran et al. 2016; Wei et al. 2014; Zanon et al. 2013).

Fungal isolates with S morphotype accounted for 6% of the examined population. As a group, these isolates produced significantly more (P <0.0001) aflatoxins in maize fermentations than A. parasiticus and isolates with L morphotype (Fig. 2, Table 4). The observed high toxigenic potential indicates that fungi with S morphotype, even at the low frequency detected, are important causal agents of aflatoxin contamination in Ghana. Several studies have implicated isolates with S morphotype as the most important causal agent of contamination in regions where dry, hot conditions occur (Cardwell and Cotty 2002; Donner et al. 2009; Ehrlich et al. 2007; Jaime-Garcia and Cotty 2010; Ortega-Beltran et al. 2015; Orum et al. 1997; Probst et al. 2010). Although isolates with S morphotype were detected in all three AEZs, it was more common in both DS and SGS, which are both hotter and drier than HF. In DS and SGS, the relatively high incidences of isolates with S morphotype in both crops coupled with both prolonged storage and dry, hot temperatures possibly accounted for the high aflatoxin levels detected (Table 2). Aflatoxin management strategies should be designed to target this highly toxigenic group of fungi within these AEZs.

Several phylogenetically divergent groups have been delineated within fungi with S morphotype using DNA-based techniques (Frisvad et al. 2005; Perrone et al. 2014; Pildain et al. 2008; Probst et al. 2012, 2014; Varga et al. 2011). Previous studies classified a group of isolates with S morphotype from West Africa as unnamed taxon SBG because those isolates uniformly produced both B and G aflatoxins (Atehnkeng et al. 2008; Cotty and Cardwell 1999; Diedhiou et al. 2011; Donner et al. 2009). On the contrary, the S morphotype found in the U.S. uniformly produce only B aflatoxins (Cotty 1997; Cotty and Cardwell 1999). In Kenya, isolates with S morphotype were identified as the primary causal agents of severe maize contamination resulting in the most ever recorded human deaths due to aflatoxicosis (Lewis et al. 2005; Probst et al. 2007). However, those Kenyan isolates are phylogenetically distinct from those from the U.S. and other regions across the globe (Probst et al. 2012, 2014).

In our study, 311 isolates were assigned to the S morphotype based on morphological and physiological characteristics. All, except nine isolates of the S morphotype produced both B and G aflatoxins; those nine isolates produced neither B nor G aflatoxins. The isolates producing both B and G aflatoxins possibly belong to the unnamed taxon SBG reported only in West African nations (Probst et al. 2014). Atoxigenicity in isolates with S morphotype has been reported only once (Horn and Dorner 1999), although in that study isolates were evaluated in chemically defined media. It is not known if that genotype failed to produce aflatoxins because of a defective aflatoxin biosynthesis gene cluster or because the media utilized was not the appropriate substrate. The nine atoxigenic isolates with S morphotype were recovered from a single maize sample from the Volta region in HF (data not shown). It is unclear whether those isolates belong to an atoxigenic lineage of the unnamed taxon SBG or to other S morphotype lineages. Molecular characterization would clarify relationships among and within this group of atoxigenic isolates and other groups of isolates with S morphotype native to Ghana and other regions of the globe.

Generally, aflatoxin B1 is the most common aflatoxin produced by aflatoxin-producing species (Mehl et al. 2012). In our study, however, 70% of the isolates with S morphotype produced atypically high aflatoxin G1 to B1 ratios in maize fermentations. Those isolates were recovered in all three AEZs (Table 4). The unusual aflatoxin production profile of certain S morphotype isolates suggests that in Ghana total aflatoxins rather than only aflatoxin B1 should be considered to determine action levels for crop aflatoxin content. A similar suggestion has been made in Malawi due to the high incidence of aflatoxin G in crops (Matumba et al. 2015).

Few studies report fungi producing higher G1 to B1 aflatoxin ratios. These include fungal communities examined in Central Europe, Africa, Asia, and the Americas (Baranyi et al. 2015; Cotty and Cardwell 1999; Ehrlich et al. 2007; Kachapulula et al. 2017; Ortega-Beltran and Cotty, unpublished). In crops, higher aflatoxin G1 to B1 ratios have been reported in naturally contaminated maize from Nigeria (Atehnkeng et al. 2008), and both maize and groundnut from Malawi (Matumba et al. 2015) and Zambia (Kachapulula et al. 2017). In Zambia, the unusual ratios were associated with A. parasiticus. In G-type producing species, differences in production of aflatoxin B1 and G1 are related to aflR to aflS expression ratio. Temperatures between 20 and 30°C favor higher expression of the aflS gene and subsequently higher biosynthesis of aflatoxin G1 as opposed to temperatures >30°C, which promote higher aflatoxin B1 biosynthesis (Schmidt-Heydt et al. 2010). However, the temperature in aflatoxin-production ability tests conducted in the current study was 31°C. It is unclear if the unusual high aflatoxin G1 to B1 ratio detected in the S morphotype fungi is dependent on temperature and/or nutritional composition of the substrate, or due to the isolates’ inherent capacity to produce higher quantities of aflatoxin G1. Noteworthy, the crop samples from which these isolates were obtained did not contain higher G1 to B1 ratios (data not shown).

Cropping systems influence proportions of Aspergillus section Flavi genotypes in any given area (Jaime-Garcia and Cotty 2010; Mehl and Cotty 2013). Nonetheless, despite differences in cropping systems in the examined AEZs, incidences of each type of fungi were similar (Table 3). There were, however, some differences in proportions of fungi between crops in certain AEZs. For example, highly toxigenic A. parasiticus isolates were detected, albeit at low frequencies, only in groundnut (Table 4), suggesting that A. parasiticus is more commonly associated with groundnut than to maize in Ghana. Similar observations have been made in other regions (Horn 2003). In general, a significantly (P < 0.05) higher association of toxigenic L morphotype (Fig. 3) and both isolates with S morphotype, and A. parasiticus was detected in groundnut than in maize (Table 3). This suggests that in Ghana, groundnut is more prone to aflatoxin contamination than maize. However, because of variability in fungal communities’ composition among years and within AEZs, aflatoxin management interventions aimed to exclude toxigenic fungi should be implemented in both crops.

This is the first comprehensive study highlighting prevalence of aflatoxin contamination in major maize and groundnut producing regions in Ghana together with both community structure of causal agents of contamination and their aflatoxin-producing potentials. Hotspot regions for aflatoxin contamination of these crops have been identified based on both environmental conditions favorable for aflatoxin contamination and high frequencies of highly toxigenic fungi, suggesting areas for preferential aflatoxin management efforts. However, studies in additional years should be conducted to determine if crops grown in these regions are perennially at risk of aflatoxin contamination. Furthermore, a fairly large collection of atoxigenic A. flavus L morphotype isolates (847 total) has been generated in this study. These atoxigenic isolates are currently being characterized to develop aflatoxin biocontrol management programs for Ghana (Bandyopadhyay et al. 2016). It is necessary to identify native, widely distributed, and competitive atoxigenic genotypes of A. flavus associated with crops and/or soils of target AEZs in Ghana. Generating knowledge on the distribution and crop association of atoxigenic L morphotype genotypes throughout Ghana will aid in selecting the best biocontrol candidates to reduce incidences and severities of aflatoxin contamination. Atoxigenic biocontrol of aflatoxins offers an economic, environmentally sound, cost-effective method of aflatoxin mitigation (Bandyopadhyay et al. 2016; Cotty 2006; Mehl et al. 2012; Wu et al. 2008). Implementing aflatoxin biocontrol managements strategies within Ghana would result in improved health, enhanced trade, increased income, and welfare of farmers and consumers.
